# Cyclodextrins: Only Pharmaceutical Excipients or Full-Fledged Drug Candidates?

**DOI:** 10.3390/pharmaceutics14122559

**Published:** 2022-11-22

**Authors:** Tamas Kovacs, Peter Nagy, Gyorgy Panyi, Lajos Szente, Zoltan Varga, Florina Zakany

**Affiliations:** 1 Department of Biophysics and Cell Biology, Faculty of Medicine, University of Debrecen, H-4032 Debrecen, Hungary; 2 CycloLab Cyclodextrin R & D Laboratory Ltd., H-1097 Budapest, Hungary

**Keywords:** cyclodextrin, inclusion complex, non-covalent interaction, cholesterol extraction, direct protein action, ion channels, Niemann–Pick type C disease, atherosclerosis, neurodegenerative diseases

## Abstract

Cyclodextrins, representing a versatile family of cyclic oligosaccharides, have extensive pharmaceutical applications due to their unique truncated cone-shaped structure with a hydrophilic outer surface and a hydrophobic cavity, which enables them to form non-covalent host–guest inclusion complexes in pharmaceutical formulations to enhance the solubility, stability and bioavailability of numerous drug molecules. As a result, cyclodextrins are mostly considered as inert carriers during their medical application, while their ability to interact not only with small molecules but also with lipids and proteins is largely neglected. By forming inclusion complexes with cholesterol, cyclodextrins deplete cholesterol from cellular membranes and thereby influence protein function indirectly through alterations in biophysical properties and lateral heterogeneity of bilayers. In this review, we summarize the general chemical principles of direct cyclodextrin–protein interactions and highlight, through relevant examples, how these interactions can modify protein functions in vivo, which, despite their huge potential, have been completely unexploited in therapy so far. Finally, we give a brief overview of disorders such as Niemann–Pick type C disease, atherosclerosis, Alzheimer’s and Parkinson’s disease, in which cyclodextrins already have or could have the potential to be active therapeutic agents due to their cholesterol-complexing or direct protein-targeting properties.

## 1. Introduction

**Figure 1:**
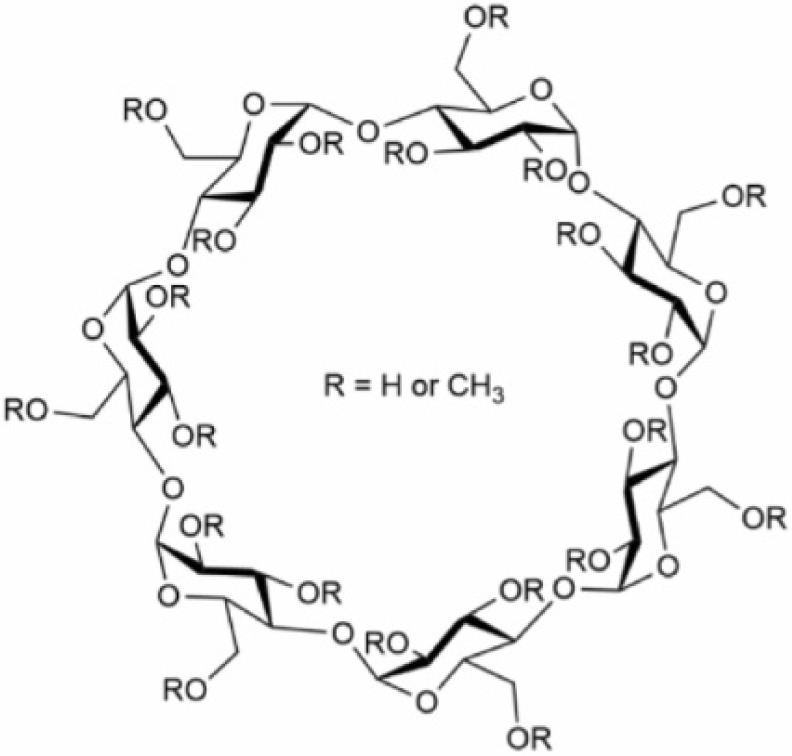
Molecular structure of randomly methylated β-cyclodextrin (MβCD). As a prototypical βCD derivative, the truncated cone-shaped structure of MβCD consists of seven α-1,4-D-glucopyranoside units and it is characterized by a hydrophobic central cavity and a hydrophilic outer surface suitable for forming inclusion complexes with hydrophobic groups in aqueous solutions. MβCD, the most commonly applied derivative for cholesterol depletion in vitro, is randomly methylated at hydroxyl groups as indicated by R groups in the figure.

Cyclodextrins (CDs) are cyclic oligosaccharides composed of six (αCDs), seven (βCDs) or eight (γCDs) α-1,4-D-glucopyranoside units with the shape of a toroidal, hollow, truncated cone. Their exterior is hydrophilic due to the OH-6 primary groups on the narrow rim and OH-2 and OH-3 secondary hydroxyl groups on the wide rim, while their cavity is hydrophobic lined with H-3, H-5 and H-6 hydrogens and O-4 ether oxygens, which gives CDs the ability to encapsulate hydrophobic molecules or moieties in their cavities (Figure 1). The glucose molecules in the ring structure are arranged rather rigidly in a ^4^C_1_ chair conformation. The molecular structure is stabilized by intramolecular hydrogen bonds between the secondary hydroxyls of the neighboring units, creating a complete belt of bonds in the case of βCD resulting in its remarkably poor water solubility. However, chemical substitution at the belt-forming hydroxyls increases aqueous solubility arising from the formation of hydrogen bonds with surrounding water molecules. A great variety of chemical modifications of CDs has been described (for example in βCD the 21 hydroxyl groups make 2^21^-1 possible combinations for substitution, and the introduction of an optically active center results in an enormous number of geometrical and optical isomers for even one type of chemical substituent) and the addition of functional groups at the hydroxyls fine tunes their size and solubility. The cavity sizes of the basic compounds are 4.7/5.3 Å, 6.0/6.5 Å and 7.5/8.3 Å for αCD, βCD and γCD, respectively, as measured as the annular diameter at the two sides of the cone-shaped molecules; however, these values can largely change depending on the number and type of additional chemical groups. The most commonly applied modified CDs include randomly methylated βCD (MβCD), 2-hydroxypropyl-βCD (HPβCD) and 2-sulfobutylether-βCD (SBEβCD) for research or therapeutic uses [1,2,3].

Complexation abilities of CDs resulting from their unique and diversified structure provide the basis for their widespread application, such as in agrochemicals, pharmaceuticals, fragrances and foods. Their biocompatibility, good tolerability, water-solubility, non-immunogenicity and resistance to degradation enable their use in pharmaceutical formulations, which is highly favorable especially in light of the newly emerging drug candidates having continuously increasing molecular mass, lipophilicity and reduced water solubility. Currently, up to 100 drug formulations contain CDs and two functionalized derivatives, HPβCD and SBEβCD, are available in FDA (United States Food and Drug Administration) and EMA (European Medicines Agency)-approved products for human parenteral use due to their favorable safety profile [1,2,4]. Recently, novel directions of CD development emerged such as design and synthesis of novel CD derivatives, CD nanosponges, covalent CD–peptide/protein conjugates, CD-based polymers formed by covalent bonds, or CD-based polyrotaxanes that can be characterized by more favorable properties compared to monomeric CDs, which include higher complexation efficiency, enhanced bioavailability, improved solubility and stability in drug formulations, better targeted delivery and release, more favorable pharmacokinetic profiles, more efficient penetration through the blood–brain barrier and even lysosomal targeting, as reviewed elsewhere [5,6,7,8].

While a lot is known about the pharmaceutical applications of CDs as excipients, their potential medical use as biologically active therapeutic agents is a more neglected area of CD research and development. Although their cholesterol-complexing ability has been known for decades, their modulatory actions in diseases characterized by pathologically relevant elevations in cholesterol levels such as Niemann–Pick type C disease, atherosclerosis, or Alzheimer’s disease have only recently been recognized. Even more scarcely documented aspects of CDs are their potential interactions with cellular proteins in spite of the fact that these compounds are often used in many peptide/protein formulations. Such potential direct CD effects on protein functions can be of substantial biological relevance considering the high CD concentrations reached when CDs are applied as vehicles of therapeutically active agents. While most recent reviews focus on the excipient actions of CDs, this review is devoted to shed light on the sovereign therapeutic relevance of CDs, which could be based on their well-known cholesterol-extracting effects and ligand-like actions on proteins that are only recently being explored experimentally but are still ignored in clinical practice. To that end, we summarize general molecular patterns of CD binding to proteins and collect the numerous examples existing in literature in which direct CD–protein interactions are revealed in detail and carry functional consequences of potential but unexploited therapeutic relevance. We further intend to emphasize the applicability of CDs as active compounds in the treatment of human disorders related to membrane cholesterol elevations, such as Niemann–Pick type C disease, atherosclerosis and neurodegenerative disorders such as Alzheimer’s and Parkinson’s diseases.

## 2. Interactions between Cyclodextrins and Lipids

### 2.1. Cholesterol Complexation by Cyclodextrins

CDs can extensively interact with a large variety of both hydrophobic and amphiphilic lipids including cholesterol, fatty acids, phospholipids, or sphingolipids by forming host-guest type inclusion complexes. The extent of encapsulation is determined by the characteristics of both the CD such as cavity size and chemical microenvironment of the cavity entrance due to substitutions, and the lipid such as chain length, presence of double bonds or polarity of the headgroup region. In general, fatty acids, phospholipids and sphingolipids show preferential complexation with the smallest αCD since their acyl chains fit tightly into its narrow hydrophobic cavity while loosely interacting with larger βCDs and γCDs. While direct interactions between CDs and fatty acids, phospholipids, or sphingolipids can be utilized in a variety of applications in food, nutraceutical or pharmaceutical industry as reviewed elsewhere [9,10], cholesterol–CD interaction is undoubtedly the most extensively studied area of lipid-CD research carrying established medical relevance, therefore, it will be in the focus of the current review.

**Figure 2:**
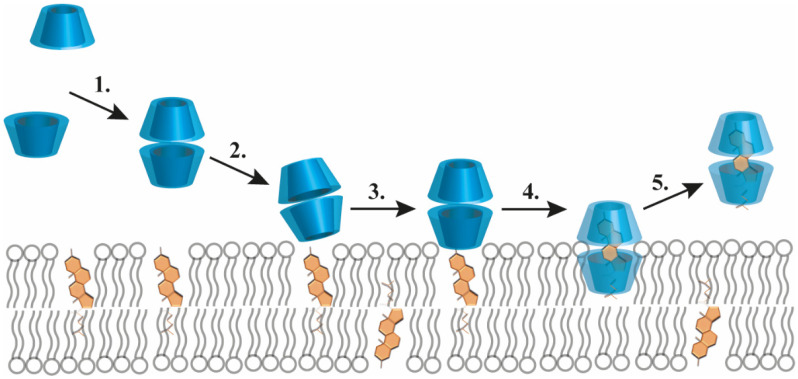
Molecular events of β-cyclodextrin (βCD)-mediated cholesterol extraction from biological membranes. The figure lists molecular events of membrane cholesterol depletion in response to βCDs determined by molecular dynamics simulation. The main steps include 1. formation of βCD dimers from monomeric βCD rings in the aqueous solution; 2. binding of βCD dimers at the bilayer-water interface in a tilted conformation; 3. reorientation of the membrane-associated βCD dimers resulting in a configuration perpendicular to the plane of the membrane; 4. gliding of a cholesterol into the hydrophobic cavity of βCD dimers; 5. desorption of the βCD dimer-cholesterol complex from the membrane [14,15].

As opposed to free fatty acids and phospho- or sphingolipids, cholesterol is complexed most efficiently with βCD and its derivatives, particularly MβCD and HPβCD [11]. Although the cavity of a single βCD is too small to shield the hydrophobic region of a cholesterol molecule from water, two stacked βCDs can provide an ideal steric fit. In a typical 1:2 (mol/mol) cholesterol:βCD complex, the almost planar cholesterol is completely encapsulated from both ends of the molecule, as its ring A and ring B are included in one βCD cavity while ring D and the side chain by the other βCD with the wider ring of the two βCDs facing each other [9]. Such a cholesterol–CD interaction can be used to manipulate cholesterol levels in biological membranes through a CD-mediated cholesterol extraction from bilayers as was demonstrated by a large number of cellular studies. In these reports, the extent of membrane cholesterol depletion was determined by various factors including the type and concentration of the applied βCD, cell type, incubation time and temperature [10,12,13]. In the original model describing the mechanism of βCD-induced cholesterol efflux, cholesterol molecules were proposed to spontaneously leave the cell membrane by an aqueous diffusion mechanism involving the initial desorption of cholesterol molecules from the membrane followed by binding to βCDs localized in the aqueous phase without binding or inserting into the bilayer [12]. In a modified version of the theory, βCDs were proposed to diffuse into the immediate proximity of the membrane so that cholesterol molecules can enter directly into the hydrophobic pocket of a βCD, without the necessity of completely desorbing through the aqueous phase [13]. According to a recently described molecular extraction model based on molecular dynamics simulations summarized in Figure 2, βCDs have a tendency to form aggregates including dimers that can bind to the membrane surface in a tilted conformation that is unable to extract cholesterol. However, further accumulation of βCD dimers on the bilayer surface stabilizes a perpendicular orientation of dimers suitable for the extraction process to occur. Once an interfacially-embedded βCD dimer is positioned above a cholesterol molecule, the latter can enter the hydrophobic cavity of βCDs rapidly, which is followed by desorption of the βCD dimer-cholesterol complex from the interface. Although βCD monomers can also bind to the membrane and capture cholesterol headgroups, they fail to induce extraction due to inadequate shielding of the whole hydrophobic cholesterol molecule. Only the last step of the process involves a substantial energy barrier and the extraction efficiency substantially depends on the lipid composition of bilayers, mainly their cholesterol content and the degree of unsaturation of phospholipids [14,15].

While empty βCDs are used to deplete membrane cholesterol, βCDs can also be pre-complexed with cholesterol and, depending on the ratio between the amounts of βCD and cholesterol in the complex, they can act as cholesterol acceptors or even donors to replenish or overload membrane cholesterol. The application of these βCD–cholesterol complexes, typically the most effective MβCD–cholesterol, can help understanding the roles of cholesterol in the modulation of cellular functions. First, repletion of cholesterol-depleted cells can revert functional alterations induced by cholesterol extraction, which can corroborate the role of cholesterol in the given process and rule out off-target βCD effects. However, the repletion procedure has to be optimized for the given experimental system to avoid effects induced by cholesterol levels far above control. Second, pre-loaded complexes can lead to cholesterol enrichment of biological membranes providing a possibility to investigate functional effects of elevated cholesterol levels mimicking human pathological conditions such as hypercholesterolemia [10,16,17,18].

Besides cholesterol, MβCD can form inclusion complexes with other sterol derivatives as well, which can be used to selectively load a given sterol into the membrane. For example, chiral analogues of cholesterol such as epi- and ent-cholesterols can be applied this way and their effects can be compared. Since these derivatives generally modify membrane biophysical parameters and lateral heterogeneity of the cell membrane in a similar manner, a stereospecific effect strongly argues in favor of modulation of protein function through direct binding [10,17,19]. Additional information about the mechanism of cholesterol-induced effects can be gained when applying MβCD complexes formed with various sterols including 7-dehydrocholesterol or 6-ketocholestanol that induce distinct changes in membrane biophysical parameters, and, therefore, can be applied to examine the possible contribution of these factors to functional effects [18,20].

### 2.2. Cyclodextrin Effects on Biophysical Parameters of Cellular Membranes

Cholesterol is a major component of biological lipid bilayers and its level substantially determines the biophysical properties of membranes including fluidity, rigidity, thickness, lateral pressure, lipid order and dipole potential. Cholesterol increases lipid order and thus reduces fluidity (i.e., increases the degree of motional constraints of macromolecules) in model membranes in the biologically relevant fluid phase, and living cells as well, due to stretching of phospholipid acyl chains and decreased average cross-sectional area per lipid molecule [21,22,23]. Cholesterol-induced decreases in membrane fluidity are also mirrored by reductions in the degree of membrane hydration (i.e., penetration of water molecules to deeper layers of membranes) [24,25,26,27]. In keeping with stretching induced in lipid acyl chains, cholesterol increases the thickness of bilayers [21,28]. In parallel, the amount of membrane cholesterol positively correlates with the interfacial elastic area expansion/compressibility moduli of bilayers implying elevated stiffness [29,30]. Furthermore, cholesterol also increases the elastic bending modulus of bilayers referring to enhanced bending rigidity [31,32], and modifies the intrinsic spontaneous curvature of membranes [33,34]. Dipole potential is an enigmatic membrane biophysical parameter, a largely positive intramembrane potential generating an immense electric field, which originates from the non-random alignment of molecular dipoles of carbonyl groups, cholesterol and water molecules at the membrane-water interface [17,35,36]. Its magnitude is determined by the lipid composition of bilayers with cholesterol being the most important determinant. The level of cholesterol shows unequivocal positive correlation with the value of dipole potential due to its intrinsic dipole moment, effects on lipid order and water penetration into the membrane [37,38]. As can be seen from the above, the level of cholesterol essentially influences the biophysical properties of cellular bilayers, which can in turn modulate the function of proteins in an indirect manner as discussed below.

**Figure 3:**
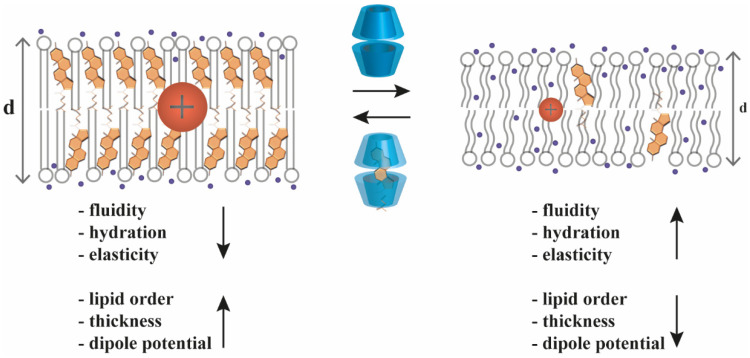
Cyclodextrin effects on biophysical parameters of biological membranes. Treatment of biological membranes with cyclodextrin (upper arrow) and concomitant cholesterol extraction results in substantial alterations of membrane biophysical parameters including increased fluidity (lower degree of motional constraints of macromolecules), hydration (penetration of water molecules represented with dark blue spheres into deeper layers of membranes) and elasticity, and decreased lipid order (represented as straightened acyl chains of phospholipids), bilayer thickness (represented as increased d distance) and dipole potential (represented as enlarged red sphere of positive charge in the central region of the membrane). On the contrary, cholesterol loading of membranes using cyclodextrins pre-complexed with cholesterol (bottom arrow) leads to opposite changes such as reduced fluidity, hydration and elasticity, and enhanced lipid order, thickness and dipole potential of cellular membranes.

Due to their cholesterol-extracting abilities, CDs can change the above-mentioned cholesterol-dependent membrane biophysical parameters (Figure 3). For example, MβCD treatment of living cells was shown to increase membrane fluidity [22,23] and hydration [25,27], and reduce the magnitude of dipole potential [20,39,40,41,42]. On the contrary, treating cells with MβCD pre-complexed with cholesterol reduced membrane hydration [27] and elevated dipole potential [20].

### 2.3. Cyclodextrin-Induced Disruption of Lipid Raft Microdomains

As can be seen above, cholesterol is an essential structural constituent of biological membranes; however, its lateral distribution is not homogenous due to its interactions with other membrane components including lipids and proteins. Preferential interactions between cholesterol and (glyco)sphingolipids, together with the active contribution of certain transmembrane proteins and the actin cytoskeleton, provide the basis for the formation of lipid rafts that are dynamic supramolecular clusters of various size characterized by elevated levels of cholesterol, phospholipids with saturated chains and (glyco)sphingolipids [17,43,44]. Various transmembrane proteins tend to accumulate in these microdomains that serve as concentrating platforms for molecules that interact with each other. In that way, raft residency facilitates the efficiency of various signaling pathways, thereby modulating a multitude of cellular functions including regulation of apoptosis, cell adhesion and migration, synaptic transmission, pathogen entry or formation of extracellular vesicles. Therefore, changes in the distribution of proteins between raft and non-raft membrane regions can lead to alterations in signaling mechanisms potentially contributing to the pathogenesis of various disorders [45,46,47,48,49].

Although cholesterol is present at higher concentrations in lipid rafts, it can also be found in non-raft regions of biological membranes. Therefore, cholesterol-complexing CDs could theoretically deplete both raft and non-raft membrane regions. Initial studies with model and cellular membranes suggested that CDs preferentially extract raft cholesterol especially in cases of short exposures and low applied concentrations, which leads to a selective removal of cholesterol from these regions eventually resulting in the consequent disruption of these ordered microdomains [10,50,51,52,53]. Subsequent reports using model giant unilamellar vesicles and molecular dynamics simulations questioned preferential cholesterol extraction from rafts and suggested that CDs rather deplete cholesterol from disordered non-raft domains of bilayers with higher efficiency. However, reduction of cholesterol levels in non-raft areas might in turn be followed by a rapid re-equilibration of cholesterol between raft and non-raft domains leading to reduced raft cholesterol levels and consequent disappearance of these microdomains [15,54]. Most probably, CDs can deplete cholesterol from both raft and non-raft bilayer regions and the efficiencies of the two processes may depend on experimental conditions such as concentration and duration of CD treatment or lipid composition of target membranes. Nevertheless, extraction from both membrane domains eventually leads to the disruption of lipid rafts in response to CDs, which can modulate the functions of transmembrane proteins localized in these regions, as discussed below.

### 2.4. Indirect Modulation of Protein Functions by Cyclodextrins via Alterations in Biophysical Parameters or Lateral Heterogeneity of Membranes

Given that molecular rearrangements associated with the activation of transmembrane proteins are mediated through the permission and cooperativity of the surrounding bilayer, lipids of the cell membrane in general, and cholesterol in particular, can actively modulate their structure and functional activity through a mixture of direct, ligand-like mechanisms and indirect effects. While the former are mediated by direct binding at cholesterol binding sites, the latter can occur via alterations in the biophysical properties or the lateral heterogeneity, i.e., the microdomain organization of cellular membranes [17].

From among effects exerted on membrane biophysical parameters, changes in membrane thickness induced by cholesterol can be highly relevant for the functional regulation of proteins, since, according to the hydrophobic mismatch theory, differences between the hydrophobic thickness of lipid constituents and transmembrane domains of proteins are energetically highly unfavorable. Thus, a larger extent of such mismatch induces adaptation mechanisms that may involve lipids such as changes in stretching of acyl chains or aggregation into preferential assemblies, for example, lipid rafts, and proteins as well. The latter include aggregation, homo- or heterooligomerization of proteins to minimize the exposed hydrophilic area, tilt of transmembrane helices or adaptation of other conformations, all of which might result in changed functional activity of the given protein. Cholesterol extraction in response to CDs and cholesterol enrichment with CD–cholesterol complexes could favor protein configurations with shorter and longer hydrophobic thickness, respectively, which can be associated with different activities [55,56,57,58].

Membrane curvature is a property of lipid bilayers strongly related to hydrophobic matching. In a membrane, when two molecules such as lipids and proteins are in the vicinity of each other, attractive and repulsive forces arise between them, which are of different type and magnitude at different depths in the membrane. As a result of these forces, there is an energetically favorable equilibrium distance between the molecules, which can be different close to the membrane-water interface and at the center of the bilayer, leading to a spontaneous curvature of the membrane. However, if the spontaneous curvature cannot be fulfilled due to steric constraints (for example because of hydrophobic mismatch) curvature elastic stress (frustration) arises. In a mutual relationship, the presence of transmembrane proteins can increase or decrease elastic stress of lipids, while the intrinsic curvature of lipids can influence the proteins. In this way, CD-induced changes in membrane cholesterol levels can modify elastic coupling between proteins and lipids, and consequently alter the stabilities of protein conformations thereby affecting their functional activity [56,58,59].

Through affecting membrane fluidity and hydration, as well as hydrophobic mismatch and elastic coupling between proteins and lipids, cholesterol has previously been consistently described to substantially modulate a large variety of structurally and functionally different transmembrane proteins including voltage- and calcium-activated large conductance potassium (BK) channels [60], nicotinic acetylcholine receptors [61], Na^+^-K^+^ ATPase [62], rhodopsin [63,64,65], metabotropic glutamate receptors [66], β_2_-adrenergic receptors [67], serotonin receptors [68,69], ErbB proteins [70] and mechanosensitive Piezo channels [71].

Through these mechanisms, i.e., increased fluidity and hydration, and reduced thickness and elastic stiffness, CD-induced cholesterol depletion was demonstrated to cause shifts in the voltage dependence of steady-state inactivation of Na_V_1.4 channels towards hyperpolarized potentials [72], reduce ATPase and pumping activity of P-glycoprotein [73], diminish ligand binding of cholecystokinin receptors [74] and enhance activation of rhodopsin [75]. When examined, cholesterol repletion or enrichment using CD–cholesterol exerted opposing effects [72,74,75]. Furthermore, by increasing membrane tension due to cholesterol extraction, CDs were suggested to act as universal activators of mechanosensitive channels [76].

Indirect effects on protein function exerted by cholesterol and CDs can also be mediated through alterations in membrane dipole potential. Since the charge distribution is typically non-uniform in proteins, and their conformational changes involve transitions of their transmembrane domains localized in the intramembrane region of the dipole potential, its associated enormous electric field can substantially modify the conformational stability of proteins [17,35,77]. In keeping with this hypothesis, the dipole potential was shown to modulate the function of bacterial ionophores [78], voltage-gated ion channels [79], Na^+^-K^+^ ATPase [80], ErbB proteins [81] and the cellular entry of cell-penetrating peptides [20,82]. Therefore, CD-induced cholesterol depletion can affect protein functions via decreases in dipole potential as shown by decreased ligand binding of P-glycoprotein [40] or serotonin receptors [39,41] in response to CDs. On the other hand, increasing cholesterol levels with cholesterol–CD complexes typically results in dipole potential-dependent changes of opposite direction [41].

A large variety of transmembrane proteins preferentially reside in lipid raft microdomains that serve as concentrating platforms for interacting molecules and are characterized by unique biophysical properties including decreased fluidity, hydration and elevated lipid order and dipole potential [22,23,25,83,84]. This unique microenvironment can differentially affect the stability of certain protein conformations and, therefore, the functional activity of proteins can be different when residing in raft or non-raft regions, as reviewed recently [17]. CD-induced disruption of lipid rafts can induce relocalization of proteins into disordered phases of cellular membranes and consequently modify their activity. Such CD effects have been described for a multitude of transmembrane proteins including various voltage-gated (K_V_) potassium channels such as K_V_1.3 [85], K_V_1.4 [86], K_V_1.5 [87], K_V_4.2 [88,89], K_V_4.2 [86], K_V_10.1 [90] and K_V_11.1 [91]. In these channels, raft localization was proposed to generally exert inhibitory effects on channel function, while a CD-mediated disruption of these microdomains rather resulted in increased activity such as elevated current amplitudes or leftward shifts in voltage dependence of activation. On the other hand, loss of raft-mediated clustering might interfere with the mostly unknown non-canonical signaling roles of ion channels [90,92]; however, this assumption remains to be proven. On the contrary, cholesterol replenishment or enrichment of the cell membrane can lead to enhanced raft partitioning, which may lead to functional changes opposed to those observed after cholesterol depletion including decreases in current amplitudes and rightward shifts of voltage dependence, as demonstrated for K_V_1.3 [18,85], K_V_1.4 [86], K_V_4.2 [89], K_V_10.1 [18] and K_V_11.1 channels [91]. Similar to K_V_s, BK channels were also found to preferentially localize into lipid rafts, and CD treatment induced their relocalization into non-raft regions, which was associated with increased current densities and leftward shifts in the voltage dependence of current activation, while cholesterol loading had opposite effects when examined [93,94,95,96]. On the other hand, disruption of caveolar microdomains in response to CD reduced functional coupling between BK channels in the plasma membrane and ryanodine receptors in the sarcoplasmic reticulum in smooth muscle cells resulting in disappearance of Ca^2+^ microdomains at plasma membrane–sarcoplasmic reticulum junctions [97]. Similarly, CD-induced disassembly of lipid rafts and concomitant relocalization of Na_V_1.8 resulted in impaired neuronal excitability and inability to conduct mechanically- and chemically-evoked depolarizations [98], while such treatment also led to the impaired signaling activity of Na^+^/K^+^ ATPase [99], TRPC1 (transient receptor potential canonical 1) [100] or TRPA1 (transient receptor potential ankyrin 1) [101] ion channels that were suggested to occur through diminished signaling platforms. These observations emphasize the importance of CD-induced effects on lateral membrane organization affecting the proper signaling function of ion channels. Piezo channels represent a mechanosensitive group of channels that are also localized in lipid rafts. CD-mediated disruption of these microdomains accompanied by a softening of the bilayer was shown to attenuate clustering and mechanosensitivity of these channels [102,103].

Besides ion channels, cell surface receptors comprise another large group of proteins that are modulated by raft partitioning and its changes induced by cholesterol removal or enrichment. ErbB proteins are the best-characterized historically prototypical members of receptor tyrosine kinases, which preferentially localize into lipid rafts that generally are thought to exert inhibitory roles on receptor functions [104]. In accordance, CD-induced cholesterol removal and consequent raft disruption was shown to enhance ligand binding affinity [105,106], subsequent receptor dimerization and clustering [106,107,108], autophosphorylation [105,107,109] and downstream signaling activation [105,110]. When examined, these effects were reverted by cholesterol replenishment using CD–cholesterol complexes [105,106,107,109]. Raft partitioning is also a common feature in G protein coupled receptors constituting the largest protein superfamily involved in practically all cellular functions. Cholesterol depletion in response to CDs affects the activity of these proteins, as CDs were demonstrated to reduce the ligand binding affinity of CXCR4 (C-X-C chemokine receptor type 4) [111], metabotropic glutamate receptors [112] and opioid receptors [113], and inhibit the downstream signaling of metabotropic glutamate receptors [114] and opioid receptors [115] in a lipid raft integrity-dependent manner. These changes were generally abolished by CD–cholesterol supplementation, underlining the importance of cholesterol-dependent membrane microdomains in the regulation of G protein coupled receptors [111,112,114].

As can be seen, due to their ability to form complexes with lipids, CDs can efficiently modulate the lipid profile of cellular membranes. Therefore, through the selective removal or loading of certain lipids, these compounds are invaluable tools for the examination of cell functions and their dependence on lipid levels. Furthermore, their application can contribute to a better understanding of the pathomechanism of diseases characterized by alterations in membrane lipid compositions and can even provide relevant therapeutic alternatives in the treatment of these disorders, as described in Section 4 of the current review.

## 3. Direct Interactions between Cyclodextrins and Proteins

### 3.1. General Mechanisms of Direct Cyclodextrin–Protein Interactions

Direct interactions between CDs and peptides or proteins were first suggested based on observations that CDs, and particularly βCD, could remove certain proteins from the cell membrane of erythrocytes [116]. While this observation was first attributed to an extrusion process resulting from CD–lipid interactions, subsequent studies confirmed the existence of inclusion complexes between CDs and amino acids, mainly βCD derivatives and hydrophobic and aromatic residues. This conclusion is in keeping with the cavity diameter of βCD compounds allowing an appropriate fit of the aromatic ring of Phe, Tyr, His and Trp (~2.5 Å in diameter between the C-3 and C-5 with an effective diameter of ~5 Å when considering hydrogens as well) into the hydrophobic moiety, while the cavity of αCDs and γCDs might enable only shallow and loose, or deep and loose inclusion, respectively [2,3]. In the first of such in vitro studies, competitive spectrophotometry demonstrated hydrophobic interactions between βCD (and to a lesser extent MβCD and HPβCD) and Phe, either when present alone or in oligopeptides in spite of steric constraints provided by the peptide backbone in the latter. Interestingly, the stability of complexes of Phe-containing oligopeptides was higher than that of the amino acid itself, which suggested that the presence of appropriate neighboring residues can contribute to the stability of the CD–peptide complex [117]. When comparing different residues, electrospray ionization mass spectrometry (ESI–MS) showed preferential CD-mediated complexation of aromatic amino acids (Trp, Phe and Tyr) over aliphatic ones (Val), with βCD more efficiently accommodating amino acids than αCD or γCD [118]. A potentiometric titration method determining binding constants between CDs and individual amino acids corroborated the complexation between aromatic Trp, Phe and Tyr (but not the more hydrophilic His) and βCD, while αCD only showed binding of Trp. In contrast, no inclusion of aliphatic amino acids (Ala, Val, Ile) was found with the exception of Leu. Dipeptides Ala-Phe and Ala-Tyr had higher affinities, indicating that the extension by a peptide moiety may result in stronger binding [119]. When examining binding between βCD and nine designed tripeptides obtained by permuting positions of two Ala with that of one Trp, Phe or Tyr using UV-visible and NMR spectroscopy combined with molecular docking analysis, these tripeptides showed stronger interactions than single amino acids, with Tyr-containing ones having the largest binding constants. The position of the aromatic side chain modulated βCD-tripeptide affinity, which highlighted the role of stabilizing interactions mediated by neighboring amino acids with the upper rim of βCD. It appeared that the hydrophobic aromatic rings form inclusion complexes with the hydrophobic cavity with the extent of embedding depending on the type and location of the aromatic group, and the flanking residues contribute to the stabilization of the complex interacting with the hydroxyl groups of βCD and aiding the right orientation of the aromatic ring in the cavity [120]. Recent molecular dynamics simulations also found that HPβCD can interact extensively with most apolar and polar but not charged side chains of individual amino acids with a special preference for the aromatic rings of Tyr, Phe and Trp. Furthermore, simulations also suggested the role of potential hydrogen bonds to HPβCD–amino acid interactions [121].

Examination of longer model oligopeptides also supported the existence of functionally relevant CD–amino acid interactions. Again, first reports emphasized the exclusive importance of aromatic amino acids. For example, according to UV absorption and fluorescence spectroscopy, the luteinizing hormone releasing hormone (LHRH) agonist deslorelin ([D-Trp^6^, Des-Gly^10^] LHRH) directly bound HPβCD via its Trp and circular dichroism spectroscopy revealed that this interaction resulted in the stabilization of its native conformation and protection from enzymatic degradation [122]. Similarly, fluorescence, circular dichroism and IR spectroscopy showed that the lone aromatic Trp residue of the melittin peptide of bee venom can also be intercalated into the cavity of HPβCD leading to an inhibition of its aggregation [123]. However, subsequent studies later recognized the importance of additional interactions such as hydrogen bonds in the formation of CD–peptide complexes. For example, electron capture dissociation high resolution tandem mass spectrometry located βCD binding sites on model peptides including substance B, bombesin, angiotensin I and II, which were formed by a variety of amino acids including Tyr, Asp, Asn, Gln, Lys, Arg, and Pro, and the importance of hydrogen bonds was emphasized instead of exclusive hydrophobic interactions with aromatic groups [124].

Based on findings obtained in these in vitro studies examining direct interactions between CDs and individual amino acids or short model peptides, a common complexation mechanism emerged according to which the CD–peptide association is mediated by a central stacking inclusion of an amino acid residue, mainly an aromatic one into the cavity of CDs, particularly βCD and its derivatives due to their size appropriate for an optimal steric fit. This model was based on results gained with individual amino acids or short simple model peptides; however, more complicated three-dimensional structures of longer peptides and proteins and the presence of a vast number of chemical groups in these molecules potentially interacting with CDs might add further levels of complexity of CD–peptide/protein associations. In these more complex structures, the stacking association between the CD inner cavity and the interacting central aromatic residue can be substantially stabilized by additional interactions including hydrogen bonds, hydrophobic and van der Waals interactions with neighboring residues. Subsequent determination of crystal structures of bacterial, fungal and plant carbohydrate-binding proteins in complex with CDs acting as substrates, competitive or allosteric regulators provided insights into these detailed molecular mechanisms of CD–protein binding, which are to be discussed in the following section.

### 3.2. Bacterial, Fungal and Plant Carbohydrate-Binding Proteins

The first group of carbohydrate-binding proteins that extensively interact with CDs are cyclodextrin glycosyltransferase (CGT) enzymes that produce CDs via intramolecular transglycosylation of α(1-4)-glucans such as amylose of starch. During the enzyme reaction, the product CD is attached to the enzyme, providing a possibility for the examination of molecular details of CD–protein bonds. X-ray crystallography of such a snapshot with a βCD derivative, S-(α-D-glucopyranosyl)-6-thio-β-CD bound in the active site of CGT from *Bacillus circulans* revealed molecular details of the CD–protein interaction. The contact was dominated by essential stacking interactions with aromatic Phe and Tyr residues (Tyr89, Tyr100, Phe183, Tyr195, Phe259), which were complemented by an extended network of direct and water-mediated hydrogen bonds involving a catalytic Asp residue (Lys47, Tyr89, Tyr97, Trp101, Asp196, His233, His327, Asp328, Asp371, Arg375 and the catalytic Asp229) [125].

CGT enzymes show high structural similarity with α-amylases acting as endoamylases, i.e., starch-hydrolyzing enzymes that can recognize and cleave α-1,4 glycosidic bonds at random positions along the starch chain. Certain members of the α-amylase enzyme family can bind and efficiently hydrolyze CDs. The structure of one such protein, α-amlyase II from *Thermoactinomyces vulgaris*, with a substrate MβCD located in its active cleft showed binding position and orientation identical to that of the S-(α-D-glucopyranosyl)-6-thio-β-CD in its complex with *Bacillus circulans* CGT. Very similar stacking interactions with aromatic residues (His202, Tyr204, Phe286, Trp356) and a network of hydrogen bonds (with Tyr45, His244, Glu354, Asp421, Asp465, Arg469) mediated the association [126]. While the *Thermoactinomyces vulgaris* α-amlyase II shows comparable affinity towards CDs and starch, the structurally similar maltogenic amylase of *Thermus* sp. *IM6501* exhibits a strong preference for CDs. X-ray crystallography of the enzyme in complex with βCD suggested that this difference originates from a domain-swapped homodimer configuration resulting in the formation of a narrow and deep active-site groove optimal for CD binding, which is distinct from the wide and shallow active-site groove of the smaller α-amylases. Furthermore, stacking interactions between an additional Trp residue and βCD were found essential for the preferential CD association [127].

CD binding was also described in exoamylases that hydrolyze outer α-1,4 glycosidic bonds in the starch chain and this association often results in the inhibition of their enzyme activity. X-ray crystallographic structures of βCD bound to exoamylases revealed similar molecular patterns of binding with the presence of stacking interactions with aromatic residues and an extensive network of hydrogen bonds and van der Waals or hydrophobic contacts with amino acids in proximity. Typically, in these structures the βCD sits at the base of the substrate-binding cleft occupying the cleft entrance and thus inhibiting catalysis by blocking substrate access to the more deeply located reaction center. Such associations were revealed between βCD and structurally homologous soybean β-amylase [128], maltodextrin-binding protein of *Escherichia coli* [129], cyclo/maltodextrin-binding protein of *Thermoactinomyces vulgaris* [130], and the granular starch-binding domain of glucoamlyase 1 of *Aspergillus niger*, that is structurally similar to the CD binding region of CGTs [131]. Debranching amylases, members of the third major group of amylases that cleave α-1,6 linkages, can also bind CDs resulting in a competitive inhibition of enzyme activity. CD-binding mechanisms of these proteins follow the same principles as demonstrated by crystal structures of complexes between βCD and various debranching amylases such as barley limit dextrinase [132], and pullulanase from *Klebsiella penumoniae* [133,134].

While molecular details of crystallographic structures of carbohydrate-binding proteins in complex with CDs showed subtle differences, CD–protein interactions were characterized by highly similar molecular patterns. In general, the association was mediated by stacking interactions leading to the inclusion of an aromatic amino acid into the cavity of the CD, which is enabled by an appropriate configuration of other residues in sterical proximity leading to the formation of a network of hydrogen bonds, hydrophobic and van der Waals interactions stabilizing the structure. Data obtained with human peptides and proteins to be described in the next sections are strongly consistent with this complexation mechanism.

### 3.3. Aggregation-Prone Human Peptides and Proteins

While the molecular details of direct CD–protein interactions are most extensively documented in carbohydrate-binding proteins of bacteria, fungi and plants, various human peptides or proteins were also reported to form complexes with CDs as well. The first group of such molecules with well-documented CD interactions is composed of aggregation-prone human peptides and proteins. Their aggregation is generally thought to occur due to a temporary exposure of hydrophobic regions to the surface of the molecule, which leads to formation of intermolecular associations to bury hydrophobic chemical groups and avoid their energetically unfavorable contacts with water molecules. By virtue of their hydrophobic cavity, CDs might place a hydrophilic “cap” on exposed hydrophobic residues that participate in intermolecular peptide-peptide interactions to prevent aggregation by sterically hindering association of monomers [2,135]. In one of the first studies providing definite proof for this hypothesis, NMR spectroscopy of non-carbohydrate-binding model proteins demonstrated that βCD could bind to solvent-exposed aromatic hydrophobic sites on the surface of chymotrypsin inhibitor 2 and S6 proteins, which might inhibit their association or influence functionally relevant conformational changes of proteins by modifying stabilities of certain configurations [135]. Accordingly, various βCD derivatives were shown to efficiently inhibit the chemically or thermally induced denaturation and aggregation of lysozyme and basic fibroblast growth factor [136]. A recent computational approach demonstrated stabilization of granulocyte colony-stimulating factor in response to βCD and HPβCD at air-water interfaces and also in bulk water by complexing of peptide residues. However, according to this study, CDs might include not only moieties of aromatic side chains but polar or even charged amino acids as well. Based on these observations, a general inclusion mechanism was suggested with the involvement of peptide backbones and solvent accessibility as a decisive factor for the interaction [137].

#### 3.3.1. Human Peptide Hormones

Besides the model compounds mentioned above, pharmaceutically or pathophysiologically relevant peptides and proteins were also shown to directly bind various CDs, mainly through their aromatic residues. A peptide exposing its aromatic amino acids even in its native form, thus being prone to misfolding and aggregation, is the human growth hormone (hGH). NMR spectroscopy showed its pronounced tendency to interact with mainly βCDs such as HPβCD and SBEβCD (and to a much lesser extent with αCD or γCD). This binding was suggested to involve H-3 and H-5 atoms on βCDs and Phe and Tyr residues on hGH, and resulted in decreased aggregation of the peptide [138].

Similar to hGH, ESI–MS showed complex formation between insulin and MβCD, and NMR spectroscopy suggested that this phenomenon can be mediated by aromatic residues (Tyr14 of the A chain and His5, His10 and Tyr26 of the B chain) leading to reduced self-association of the peptide [139]. Subsequent NMR studies demonstrated that βCD can bind insulin at these specific solvent-exposed aromatic Tyr and Phe sites (TyrA14, PheB1, TyrB16, PheB25). In this study, four interaction sites on monomeric insulin and one interaction site per insulin molecule in its dimeric form were detected, preferentially stabilizing the monomeric form and thus acting against aggregation [135]. As shown by NMR spectroscopy, HPβCD can also bind insulin, and while the largest chemical shift changes were found at the previously proposed Tyr and Phe residues (TyrA14, TyrA19, PheB1, TyrB26), two additional His (HisB5, HisB10) were also suggested to contribute to the complexation with this more hydrophilic CD [140]. Molecular docking analysis between insulin and various CDs corroborated complex formation with native CDs, MβCD, HPβCD and SBEβCD as well, which was mediated by inclusion of the previously described aromatic sites (PheB1, HisB10, TyrB16), and additional non-aromatic side chains (LeuA13, LysB29, ThrB30), and these complexes were stabilized by hydrogen bonds with a variety of other residues [141].

#### 3.3.2. Amyloid-Forming Peptides and Proteins

Anti-aggregative effects of CDs can be highly relevant for cellular aggregation-prone peptides and proteins that, by entering the so-called amyloid state, can form elongated fibers with spines consisting of many-stranded β-sheet structures. The process is suggested to be mediated through the exposure of backbone amide groups. Accumulation of such amyloid-state fibrils can be toxic and may lead to a great variety of human disorders such as Alzheimer’s, Parkinson’s or prion diseases, characterized by the assembly of amyloid β (Aβ) peptides, α-synuclein and abnormal prion proteins, respectively [142]. CD-mediated shielding of exposed hydrophobic groups of these molecules might interfere with the aggregation process. Consistently, ESI–MS demonstrated that βCD can form complexes with Aβ peptides, proposedly via their Tyr and Phe amino acids, which might prevent their fibrillation and diminish substantially their neurotoxic effects [143]. Analysis of CD spectra of Aβ(12-28) in the presence and absence of CDs demonstrated that βCD (but not αCD or γCD) inhibited the conformational transition of the peptide from random coil to the aggregation-prone β-sheet structure, which, according to NMR spectroscopy, was mediated by insertion of Phe19 and Phe20 to the inner CD ring from the broad side. Chemical shift changes in the methyl groups of Val18 of the peptide suggested that this residue can also play an important role in the stabilization of the interaction [144]. Subsequent NMR studies using Aβ(1-40) and Aβ(12-28) revealed two βCD binding sites, at Phe19 and/or Phe20 and Tyr10 with the two peptides showing similar affinities. Emphasizing the role of Phe residues, the Aβ(12-28)Gly19Gly20 variant did not bind βCD, while the Aβ(12-28) fragment did not interact with αCD or γCD, which supported the requirement of the good steric fit between the CD and the aromatic rings of Phe and Tyr [145]. In a subsequent study, a βCD dimer, consisting of two βCD monomers connected by a flexible pentaethylene glycol-based linker, showed increased affinity towards Aβ(1-40) in similar measurements, which also revealed additional potential interactions with His residues of the peptide. Both the βCD monomer and particularly the dimer affected Aβ aggregation and transmission electron microscopy showed altered structural morphology of the aggregates with potentially different neurotoxic activities [146]. When examining interactions between HPβCD, a physiologically more relevant CD, and Aβ(1-42), molecular docking analysis and molecular dynamics simulations confirmed the role of Phe19 in HPβCD binding; however, additional non-aromatic residues (Lys16, Ala21, Ile31 and Met35) were reported to contribute to complexation. In addition, supporting the biological relevance of this interaction, HPβCD treatment inhibited peptide self-aggregation mainly by impeding elongation and lateral association of peptide oligomers, prevented structural transition into β-sheet structures and reduced Aβ-induced toxicity. Once the inner hydrophobic cavity of HPβCD was blocked by a small hydrophobic molecule, the compound lost these protective abilities [147].

CDs, besides binding short Aβ peptides and thus interfering with their fibrillization, can also directly inhibit association of longer amyloid-forming polypeptide chains. For example, according to in vitro experiments, βCD bound to PrPC prion proteins and inhibited their conversion to the abnormal PrPSc isoform. In scrapie-infected neuroblastoma cells, βCD and MβCD treatment resulted in largely reduced cellular levels of the pathogenic PrPSc, while αCD and γCD showed much lower antiprion activity [148]. Furthermore, in transmission electron microscopy and size exclusion chromatography experiments βCD, both when applied alone or in a synergistic combination with curcumin, resulted in reduced aggregation and even dissolution of pre-formed aggregates of α-synuclein. Secondary structural analysis showed that βCD reverted α-syunclein aggregates back to the native unstructured conformation of the protein [149]. Similar synergistic anti-aggregative and disaggregating effects were found between βCD and naturally occurring polyphenols (curcumin, resveratrol, baicalein and (–)-epigallocatechin gallate) even in the presence of macromolecular crowding agents mimicking more (patho)physiological conditions, and these effects were associated with impeded cellular toxicity of the prefibrillar α-synuclein aggregates on a mouse neuroblastoma cell line [150].

As can be seen, CD interaction mechanisms of human peptide hormones and amyloid-forming proteins are similar to those described above for model oligopeptides, and usually involve inclusion of an aromatic residue. In most cases, the connection is further strengthened by hydrogen bonds and hydrophobic or van der Waals interactions with nearby amino acids. The CD–peptide interaction typically results in decreased aggregation tendency of the peptides, in some cases even dissolution of aggregates already formed, which can be favorably utilized in the medical practice as discussed in Section 4.1 and Section 4.2.

### 3.4. AMP-Activated Protein Kinase

**Figure 4:**
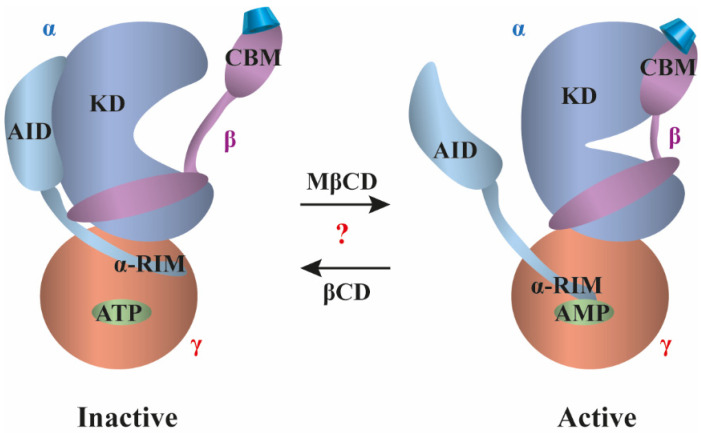
Controversial cyclodextrin-induced effects on the activity of AMP-activated protein kinase. In the inactive state (on the left), the autoinhibitory domain (AID) of the α subunit (blue) binds the kinase domain (KD) and arrests it in an inactive, open conformation. In this configuration, the carbohydrate-binding module (CBM) of the β subunit (purple) is dissociated from the KD, and the γ subunit (red) binds ATP. In the active state (on the right), AMP induces binding of the nucleotide sensor loop (α-RIM) of the α subunit to the AMP-binding site on the γ subunit. This in turn pulls the AID away from the inhibitory interaction with the KD. AMPK can also be activated by binding of compounds to the CBM inducing its association with KD, which helps to stabilize the closed, active conformation of KD. CDs (blue truncated cones) were proposed to modulate the activation by direct binding to CBM. While βCD-CBM was shown to stabilize the inactive state [152], MβCD treatment resulted in increased CBM-KD association leading to AMPK activation [155]. Elucidation of the functional effects of CDs and the mechanisms of their actions would facilitate further progress of CD application in Niemann–Pick type C disease, and possibly in other human AMPK-related pathological conditions, such as diabetes, obesity, and cancer.

AMP-activated protein kinase (AMPK) is an evolutionarily conserved metabolic stress sensing protein kinase functioning as a critical focal point for whole-body and cellular mechanisms to maintain energy homeostasis, and therefore a promising drug target for the treatment of diabetes, obesity, and cancer. AMPK is typically activated by a drop in the ratio of ATP to AMP/ADP reflecting energy stress, which promotes ATP production by increasing the activity or expression of proteins involved in catabolism while conserving ATP by switching off biosynthetic pathways [151]. It is a heterotrimeric protein complex with an α subunit containing the serine/threonine kinase domain (KD), an adenine nucleotide sensor loop (α-RIM), a kinase activation loop and an autoinhibitory domain (AID). The β subunit acts as a subcellular targeting subunit and a molecular scaffold responsible for membrane binding while having a glycogen-binding domain, and the γ subunit with two pairs of AMP/ADP/ATP-binding cystathionine β-synthase-like (CBS) domains sensitively detects shifts in the AMP/ATP ratio (Figure 4) [152]. X-ray crystallographic examination of the glycogen-binding domain of the β subunit similar to the amylase domain structures described above in Section 3.2 revealed direct interactions with five glucose units of βCD in a carbohydrate-binding module (CBM). In the structure, the βCD was held in a pincer-like grasp with two tryptophan residues cradling two glucose units and a leucine residue piercing the ring of βCD. The first glucose moiety of AMPK (G1) was involved in stacking interactions with Trp100 and the O2′ and O3′ atoms formed hydrogen bonds with Asn150, while G2 was stacked against Trp133 and formed a hydrogen bond with Lys126. G7 participated in a water-mediated hydrogen bond with Gln124. In addition, Leu146 and Thr148 formed hydrophobic interactions with the G7-G1-G2 sugar units, and the O2′ and O3′ atoms of G4 and G5 created hydrogen bonds with Gln145 and Leu146. As a result, similarly to that observed in carbohydrate-binding proteins described in Section 3.2, the AMPK-βCD association was mediated by hydrophobic stacking interactions between the sugar and the aromatic side chains of the protein, and a network of direct and water-mediated additional hydrogen bonds and hydrophobic interactions between sugar units and protein residues [153]. Supporting the functional relevance of this interaction, in a cell-free assay, similarly to glycogen, βCD binding to the glycogen-binding domain resulted in an allosteric inhibition of AMPK enzyme activity with a half-maximal effect at 1.6 mM, which was abolished by mutating Trp100 and Trp133 residues involved in βCD-AMPK binding [154]. Subsequent studies using X-ray crystallography to compare low-resolution structures of non-phosphorylated and active phosphorylated holo-AMPK bound to AMP and βCD, and luminescence proximity assay revealed molecular details of AMPK activation. According to the suggested model, the AID binds to the KD arresting it in an inactive open conformation. In the AMP-bound active state, the AID rather interacts with the α-RIM, which is induced by AMP binding to CBS-3, thereby pulling away the AID and releasing inhibition of the KD. ATP counteracts activation by competitively inhibiting AMP binding and also by destabilizing AID-α-RIM interaction (Figure 4 on the left). This process can be dynamically modulated by the CBM of the glycogen-binding domain (capable of βCD binding) that is suggested to bind the kinase domain in the active configuration and become dissociated in the inactive state. This association can be modulated as phosphorylation enhances, while the presence of glycogen or 2 mM βCD destabilizes the interaction. Consistently, mutation of the crucial Trp residue abolishes the effects of glycogen and βCD [152].

Cellular thermal shift assay and isothermal dose–response fingerprint experiments further showed the association between AMPK β subunits and a CD derivative, MβCD. However, examination of the functional consequences of this association revealed that 100 µM MβCD in fact resulted in increased phosphorylation of AMPK α subunits and elevated phosphorylation of substrates referring to AMPK activation (Figure 4 on the right). Consistent with the activation of AMPK and its involvement in the upstream regulation of autophagy, this led to a restoration of impaired autophagy flux in various cellular models of Niemann–Pick type C disease. Molecular docking analysis confirmed the molecular details of binding and the involved AMPK amino acids proposed by earlier studies, as key hydrogen bonds with residues Lys126 and Asn150, and aromatic stacking interactions with Trp100 and Trp133 were conserved. However, when comparing MβCD binding to β1 and β2 subunits, slight differences were observed in the conformation of Trp100 and Leu146, two residues substantially involved in CD interactions. It was suggested that the degree and localization of CD substituents such as methylation in MβCD might promote or impair conformational shifts, and these effects can be distinct in different β subunits [155]. Considering these findings and the widely accepted potential role of another CD derivative, HPβCD, in Niemann–Pick type C disease therapy as discussed in Section 4.4, further understanding of AMPK-CD binding and its functional consequences depending on the degree and localization of CD substituents can be highly relevant in diseases associated with AMPK.

### 3.5. Bacterial Pore-Forming Proteins

Besides professional carbohydrate-binding enzymes, pore-forming proteins comprise the second major group of proteins that were previously described to extensively interact with CDs through direct binding. Such interactions were first proposed by studies examining the possible interactions between *Staphylococcus aureus* exotoxin α-haemolysin and CDs. Due to their size and steric characteristics, CDs were hypothesized to comfortably fit inside the pore of the heptameric α-haemolysin given that the outer diameter of CDs (12.7–14.0 Å, 14.6–15.5 Å and 17.6–17.5 Å for αCD, βCD and γCD, respectively) is comparable to the narrowest internal diameter (~14 Å) of the pore at the region of the Met113 residue. Consistently, planar bilayer recordings obtained with patch-clamp revealed that βCD can enter the channel and induce reversible partial blocks of the ionic current even at micromolar concentrations. The blocking kinetics were consistent with a single binding site inside the lumen of the channel and molecular modeling suggested that βCD can bind to the restriction site, which comprises Met113, Lys147 and Glu111 residues [156]. While the average residency time for the βCD in the lumen of the wild-type α-haemolysin pore was relatively short, site-directed mutagenesis showed that mutation of Met113 to Asp, Asn, Val, His, Phe or Tyr resulted in 10^4^-fold increases in occupancy time due to reduced dissociation rate constants without any changes in the association. Remarkably, the side chains of the latter six amino acids bear little resemblance to one another, which suggested the co-existence of alternative βCD binding modes [157]. Consistent with this hypothesis, single-channel electrical recording, protein engineering including unnatural amino acid mutagenesis, and high-resolution X-ray crystallography revealed different binding modes for three groups of tight-binding mutants that comprise at position 113: (i) hydrogen-bonding amino acids Asp or Asn, and possibly His; (ii) aromatics Phe, Tyr, and possibly His, and more weakly Trp; and (iii) the β-branched amino acid Val. In homoheptamers formed by Met113Asn, a representative mutant of the first group, primary hydroxyls on the top of the CD ring face the trans entrance of the pore. In each glucose unit of the βCD, the secondary 2-hydroxyl is hydrogen bonded to the side-chain amide of an Asn113, while the 3-hydroxyl is hydrogen bonded to the ϵ-amino group of Lys147 that is also bound to the carbonyl of Asn113 in a bifurcated manner. In Met113Phe, a representative of the second group, the βCD is in the opposite orientation as secondary hydroxyls on the bottom of the CD ring face the trans entrance and each 6-hydroxyl group is in stacking CH-π interactions with the aromatic Phe113 side chain. The existence of the above two distinct binding mechanisms was corroborated by experiments with α-haemolysin heteroheptamers, which showed that while in heteromers containing WT (Met113), Met113Asn and Met113Phe subunits, replacement of the Met gradually increased affinity for βCD, heteromers formed from similar numbers of Met113Asn and Met113Phe subunits can bind βCD only weakly confirming the “opposing” effects of the two residues. The α-haemolysin with Val113 can bind βCD in a third way, as in heteromers with Met113Val, both Met113Phe and Met113Asn reduce the affinity of the pore for βCD excluding both stacking CH-π and hydrogen bonds. It was suggested that Val side-chains interact with the sides of the glucose ring, which might occur in one or both orientations of the CD ring [158]. βCD lodging into the channel lumen of α-haemolysin can in turn alter the ion selectivity of the transmembrane pore as the originally weak anion selectivity is substantially enhanced by βCD in various mutants of the channel [159]. Molecular dynamics free energy simulations and potential of mean force calculations subsequently showed that the βCD can affect selectivity through locally reducing the pore radius and causing a partial desolvation of ions and affecting the orientation of nearby charged residues, mainly the ring formed by positively charged Lys147 side chains. These changes lead to an increased electrostatic interaction between the ion and the channel due to a reduction in dielectric shielding by the solvent, resulting in augmented anion selectivity [160]. Besides α-haemolysin, the function of other bacterial pore-forming proteins can also be modulated by CDs. For example, CymA of the outer membrane component of the CD uptake and metabolism system of *Klebsiella oxytoca* can also directly bind various CDs including αCD, βCD and γCD at a binding site located inside the pore of the channel, which results in a dose-dependent block of ion transport [161].

### 3.6. Human Pore-Forming Proteins

#### 3.6.1. Connexins

Pore-forming proteins of eukaryotic cells can also directly interact with CDs and these protein–CD associations were demonstrated to affect channel function as summarized in Table 1. In the first of such reports, measurements of urea and sucrose permeation using transport-specific fractionation identified CDs as reversible pore blockers for connexin channels composing gap junctions. In these experiments, purified rat or mouse connexin-32 (Cx32) and/or connexin-26 (Cx26) proteins were reconstituted into unilamellar phospholipid liposomes not containing cholesterol, and CD treatment typically in the range of 5–20 mM resulted in a reversible complete block of conductance. The characteristics of CD-induced inhibition changed as a function of the size of the CD relative to the pore diameter, as homomeric Cx32 channels having a wider limiting diameter were completely blocked by the smaller αCD and βCD according to a step-change nature of the block-concentration relation, while the larger γCD exerted an effect in a graded fashion with increasing CD concentration. Similarly, when the narrower homomeric Cx26 channels were examined, with increasing size of CD, the block changed from steep in the case of αCD to gradual in response to βCD, while the largest γCD was without substantial effect. CD effects on Cx26/Cx32 channels were intermediate in keeping with its limiting pore diameter between that of homomeric Cx32 and homomeric Cx26. These results were explained by a simple homogenous step-like block occurring via CD entry into the pore lumen and occlusion of the permeability pathway resulting from a perfect fit of smaller CDs into the pore (as for Cx32-αCD/βCD and Cx26-αCD complexes). However, if the external width of a CD exceeds the minimal pore width of connexin channels, the imperfect fit of CD can lead to a heterogeneity of less well-defined ligand–channel interactions with an imperfect lodging into the pore and/or sideways in multiple configurations, which is mirrored by the complex, graded inhibition (for Cx32-γCD and Cx26-βCD interactions). On the other hand, a too large CD can simply fail to enter the pore thus not cause any inhibition (as in the case of Cx32-γCD). These results suggested that, similarly with bacterial pore-forming proteins, in connexin channels the pore is the primary site of CD action. This hypothesis was also supported by potentiation of the block when organic analytes were sequestered in the hydrophobic interior of CDs [162].

#### 3.6.2. GABA_A_ Receptor

While direct CD effects on channels are most extensively described in non-selective pore-forming proteins, these compounds can also interact with mildly or highly selective ion channels as well. Patch-clamp measurements on rat cultured hippocampal neurons and model simulations demonstrated that βCD modulated the function of GABA_A_ (γ-aminobutyric acid type A) receptors, anion-selective ligand-activated channels responsible for neuronal inhibition in the adult brain (Table 1). While CDs generally inhibit channel function, βCD was found to increase GABA_A_ receptor responses to ultrafast GABA applications, which was accompanied by a profound deceleration of current deactivation kinetics by slowing down the unbinding rate of the ligand and a decreased rate and extent of desensitization reflecting strong alterations in conformational transitions. βCD effects were attributed to direct interactions since the compound was applied at low concentrations (0.15–1.5 mM) at which depletion of membrane cholesterol is expected to be minor and changes were fully reversible in the given patch after an incubation of 2 min in the absence of βCD [163].

#### 3.6.3. TASK Ion Channels

**Figure 5:**
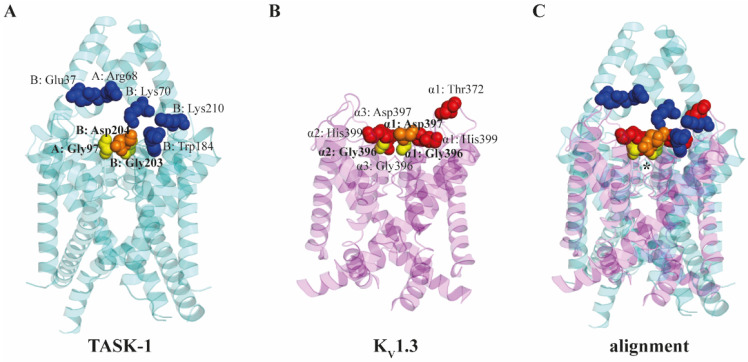
Comparison of extracellular MβCD binding sites of TASK-1 and K_V_1.3 ion channels identified by molecular docking analysis. (**A**) For TASK-1 (PDB 6RV2) amino acid residues 1 to 257 of chain A, and residues 1 to 261 of chain B are displayed (cyan). (**B**) For K_V_1.3 (PDB 7EJ1) pore-forming S5 and S6 helices are visualized for each subunit (magenta) between residues 340 and 440. (**C**) Structural alignment between TASK-1 and K_V_1.3 ion channels were performed using PyMol. The region of the selectivity filters is indicated by an asterisk. Slight differences between the orientation of helices of the two channels can be attributed to the fact that the K_V_1.3 is with an open activation gate, while TASK-1 is in a closed conformation. Amino acids contributing to the MβCD binding are indicated by red spheres for K_V_1.3 [42] and blue spheres for TASK-1 [164]. Gly396 (yellow, bold) and Asp397 (orange, bold) residues of the K_V_1.3 subunit and Gly396 (yellow, bold) of the neighboring subunit are identical with Gly203 (yellow, bold) and Asp204 (orange, bold) of the B chain, and Gly97 (yellow, bold) of the A chain of TASK-1 as revealed by the structural alignment.

A recent study [164] found that MβCD directly blocked TASK(TWIK-related acid-sensitive K^+^ channel)-1 and TASK-3 channels, two members of the two-pore-domain potassium channels, which play central roles in modulating neuronal excitability in the central nervous system by providing voltage-independent background potassium currents [165]. As demonstrated by patch-clamp measurements in rat primary cultured cerebellar granule neurons and HEK293 cells heterologously expressing these channels, 5 mM of MβCD or non-cholesterol-depleting αCD reduced currents of TASK-1 and TASK-3 channels and the heterodimer TASK-1/TASK-3 by ~40%, while the cholesterol-depleting agent filipin III exerted no effects, suggesting a direct blocking mechanism independent of cholesterol complexation (Table 1). MβCD exerted no inhibitory effects on TWIK-1 (tandem of pore domains in weak inward rectifier K^+^ channel) and TRESK (TWIK-related spinal cord K^+^ channel) currents. Consistently, molecular docking analysis identified residues potentially interacting with MβCD and αCD in the extracellular cavity close to the entry of TASK-1 channel, and another independent one in the intracellular cavity of the pore. The interacting amino acids in the most favorable binding mode at the extracellular site are Arg68 and Gly97 in the A chain and Glu37, Lys70, Trp184, Gly203, Asp204 and Lys210 in the B chain (Figure 5A). Both the extracellular and intracellular binding sites are organized by a network of hydrogen bonds and hydrophobic interactions in a configuration similar to that described in Section 3.1.

#### 3.6.4. K_V_1.3 Ion Channel

Consistent with the pore-blocking ability of CDs in TASK channels [164], a recent study revealed that MβCD can bind the K_V_1.3 ion channel and inhibit its current in a cholesterol-independent, ligand-like manner [42]. This channel has structural properties and gating mechanisms prototypical for most members of the voltage-gated potassium channel (K_V_) superfamily, and pathophysiological relevance in various autoimmune and neurodegenerative disorders [166]. In this study [42] patch-clamp measurements demonstrated that MβCD dose-dependently and partially reversibly reduced K_V_1.3 currents (5 mM MβCD reduced currents by ~40%). The inhibition was apparent within 15 s and it was completed in 90 s (Table 1). Notably, according to time-resolved flow cytometry examination of the extraction of exogenously loaded cholesterol, no significant changes in membrane cholesterol level occurred within this time frame. In addition, non-cholesterol-complexing per(3,6-anhydro)-α- and -γCDs characterized by an inverted structure with a hydrophilic interior and a hydrophobic outer surface more potently decreased K_V_1.3 currents. On the other hand, cholesterol-depleting HPβCD and HPγCD, and non-cholesterol-depleting per(3,6-anhydro)-βCD did not inhibit K_V_1.3. These findings strongly suggested a cholesterol extraction-independent ligand-like CD action since it has been described in the literature that MβCD-induced cholesterol depletion increases K_V_1.3 currents [85]. Furthermore, CD-induced effects on K_V_1.3 showed no statistically significant correlations with alterations in membrane fluidity, hydration or lipid order further arguing against cholesterol-mediated mechanisms [42]. In the same study, in silico molecular docking analysis between MβCD and K_V_1.3 supported potential direct binding of MβCD to the extracellular orifice of the pore domain. Multiple binding modes were recorded, which identified a common pattern of direct interactions mainly organized by His399, a residue representing a molecular target for pore-blocking tetraethyl–ammonium [167] and a variety of scorpion toxins [168]. The basis of interaction is provided by a hydrogen bond formed by His399, which is further strengthened by a network of hydrogen bonds and hydrophobic interactions with Gly396, Asp397 and Thr372 of the same subunit and stabilized by contacts with His399 and Gly396 of one neighboring subunit, and Gly396 and Asp397 of the opposing subunit (Figure 5B) [42].

When comparing these molecular docking results with those obtained in TASK-1 [164], significant overlap can be observed between MβCD binding sites in the extracellular regions of their pore domains as indicated in Figure 5C. Comparing the two most favorable binding modes identified in the channels, we found that Gly396 and Asp397 residues of the K_V_1.3 subunit containing the crucial His399, and Gly396 of the neighboring subunit are identical with Gly203 and Asp204 of the B chain, and Gly97 of the A chain of TASK-1. Besides these identical residues (yellow and orange, bold), the other highlighted amino acids (red in K_V_1.3 and blue in TASK-1) involved in binding also suggest a highly similar extracellular MβCD binding pocket in the pore domains. While in the case of TASK-1, an intracellular MβCD binding site was also described, in K_V_1.3 the possibility of such an intracellular site was ruled out based on the prompt current-inhibitory effect (significant current decrease after ~15 s for K_V_1.3 vs. ~80–100 s for TASK-1). The fact that these structurally different channels share similar binding sites for MβCD proposes that CDs could possibly bind to a variety of other ion channels as well, and consequently modify their ionic currents, which is worth being investigated in the future in parallel with mutagenesis studies to confirm the functional relevance of the proposed identical binding residues.

## 4. Potential Clinical Applicability of Cyclodextrin Interactions with Cholesterol and Proteins

### 4.1. Cyclodextrins as Anti-Aggregative Excipients in Peptide and Protein Drug Formulations

In the last decades, a continuously growing number of therapeutic peptide and protein drugs became available to combat a variety of human diseases. However, the application of such peptide- and protein-containing formulations is hampered by various factors, including chemical, enzymatic and physical instability, poor absorption through biological membranes, rapid plasma clearance and immunogenicity. Among these, protein aggregation can be considered the most problematic, as it can happen at almost any stage of manufacturing, processing, storage, shipment and administration, and it can largely influence the other above-mentioned factors. These obstacles, particularly the unwanted aggregation, can be at least partially overcome with the use of adjuvants and CDs were shown as attractive alternatives for this purpose by acting as solubilizers, stabilizers, artificial chaperone mimics and absorption enhancers for a variety of substances [1,2,4,7,8,169].

Since peptides and proteins are generally too large to be wholly included in the cavity of CDs, these beneficial effects are rather mediated by the formation of inclusion complexes with solvent-accessible hydrophobic amino acids of peptide chains. Depending on the exact molecular arrangement, masking of these temporarily exposed hydrophobic residues by CDs may in turn influence the conformation, aggregation, folding, degradation, surface adsorption and, consequently, the functional activity of peptides. For example, inclusion of exposed hydrophobic residues can prevent their intermolecular interactions that are typically mediated by these amino acids, thereby providing the opportunity for an aggregation-prone partially unfolded intermediate to fold back to its native, functionally active form, protecting it from aggregation or adsorption [2,117,135].

Consistent with this hypothesis, mainly βCD and its derivatives such as MβCD, HPβCD and SBEβCD were found to reduce aggregation tendency of pharmaceutically relevant peptides and even through interaction with aggregated/denatured forms to induce their natural refolding as previously shown for insulin [139,170], hGH [138,171] or granulocyte colony-stimulating factor [137].

While CDs are invaluable tools for the pharmaceutic formulations of peptide and protein drugs, such applications are thoroughly reviewed elsewhere [2,169] and, therefore, their detailed description is beyond the scope of the current review. Thus, in the next subsections, we limit our discussion to a short analysis of potential therapeutic effects of CDs in human pathological conditions.

### 4.2. Cyclodextrins as Active Anti-Aggregative and Cholesterol-Depleting Agents in Human Amyloid-Related Neurodegenerative Diseases

The favorable anti-aggregative effects of CDs can be utilized in human amyloid-related diseases characterized by an accumulation of aggregation-prone cellular peptides and proteins capable of forming neurotoxic amyloid fibrils [142]. Alzheimer’s disease is the most common neurodegenerative disorder that eventually leads to progressive dementia and it is casually linked to the extracellular deposition of aggregated fibrils built up by Aβ peptides [172,173]. CDs can interfere with various steps of Alzheimer’s disease pathogenesis as reviewed recently [174]. As described in Section 3.3.2, these compounds can directly bind Aβ peptides, inhibit their aggregation and even induce altered morphology and break-up of pre-formed amyloid fibrils leading to reduced cytotoxicity [144,146,147]. Besides these anti-aggregative effects, lipid raft disruption is also highly relevant for potential favorable actions of CDs in Alzheimer’s disease. Aggregation-prone Aβ peptides are produced from the amyloid precursor protein (APP) in the amyloidogenic pathway by sequential proteolytic degradation catalyzed by β- and γ-secretase enzymes. Alternatively, APP is cleaved by α-secretase in the nonamyloidogenic pathway that prevents amyloid plaque formation. The two pathways are separated in space since the nonamyloidogenic cleavage happens in non-raft domains of the cell membrane, while amyloidogenic hydrolysis occurs in the endolysosomal compartments after lipid raft-mediated endocytosis from the cell surface [175,176]. In cellular models of the disease cholesterol extraction and the consequent lipid raft disruption in response to MβCD consistently reduced Aβ production that was accompanied by elevated levels of the neuroprotective products of nonamyloidogenic cleavage [177,178,179,180,181,182,183]. These effects were attributed to decreased proximity between elements (lipid rafts, APP, β- and γ-secretase and endosomes) of the amyloidogenic pathway [179,180,182,184,185]. Cholesterol–MβCD treatments induced opposite effects [181,184,185,186]. In keeping with favorable effects of cholesterol depletion in cellular studies, in vivo experiments demonstrated that cholesterol extraction with well-tolerated HPβCD alleviated clinical symptoms, histopathological alterations and autophagosomal–lysosomal abnormalities accompanied by enhanced clearance of Aβ peptides in animal models of Alzheimer’s disease [187,188,189,190]. Based on the promising results obtained in in vitro and in vivo studies, a randomized, placebo-controlled, double-blind phase 2 clinical study was launched recently to assess the safety, tolerability, and potential efficacy of intravenous HPβCD infusion (500–1000 mg/kg every 28 days) in early Alzheimer’s disease (NCT05607615).

Parkinson’s disease, the second most frequent neurodegenerative disease, is pathogenically coupled to the misfolding and aggregation of α-synuclein resulting in neuronal loss especially in the dopaminergic neurons of substantia nigra [191,192,193]. As described in Section 3.3.2, in vitro experiments demonstrated that βCD can inhibit aggregation of α-synuclein and even dissolve its pre-formed aggregates [149], and cellular studies showed that this is accompanied by reductions in the toxicity of prefibrillar α-synuclein aggregates [150]. Furthermore, MβCD was found to lower α-synuclein accumulation in cellular and animal models of the disease [194]. While this effect was suggested to occur through cholesterol extraction, a subsequent study suggested an alternative mechanism of action by demonstrating that in a cellular Parkinson model 1 mM HPβCD activated TFEB (transcription factor EB), a major regulator of lysosomal functions. This resulted in a promotion of autophagic clearance of aggregated α-synuclein, suggesting potential beneficial CD effects via activation of the autophagy–lysosomal pathway [195].

### 4.3. Cyclodextrins as Active Cholesterol-Extracting Agents in Atherosclerosis

Atherosclerosis, a major cause of cardiovascular mortality worldwide, is caused by the subendothelial accumulation and sclerotic aggregation of cholesterol and other lipids in the wall of arteries and formation of macrophage-derived foam cells leading to chronic inflammation and life-threatening cardiovascular events. Considering the central role of cholesterol in the pathogenesis of the disease, cholesterol-extracting CDs can be regarded as potential therapeutic agents targeting various steps of the pathogenesis, as reviewed recently [174]. In accordance, using cellular models of the disorder various CDs, including βCD, MβCD and HPβCD were found to effectively induce cholesterol efflux from cells with elevated cholesterol levels [12,196,197,198]. CDs can also reduce elevated cellular levels of 7-ketocholesterol and other oxysterols, major products of cholesterol oxidation that accumulate in plaques and play important roles in disease pathogenesis [199]. CDs are able to favorably influence the inflammatory components of atherosclerosis as well by reducing adhesion of immune cells to endothelial cells by decreasing expression of cell adhesion molecules of the latter [200], limiting enhanced proliferation and cytokine secretion of activated lymphocytes [201], modulating cholesterol crystal-induced complement activation, reactive oxygen species production and proinflammatory cytokine and chemokine secretion of immune cells [202], and inhibiting foam cell formation from macrophages [203,204]. While all these effects induced by CDs typically applied at concentrations between 1 and 10 mM were previously attributed to cholesterol complexation, a potential contribution of direct actions on proteins involved in the regulation of cellular metabolism or pro- and anti-inflammatory cascade pathways has not been examined yet.

In vivo studies also corroborated the potential use of cholesterol-complexing CDs in atherosclerosis. In an early study, intravenous or parenteral HPβCD reduced serum cholesterol levels and aortic atherosclerotic lesions in rabbits with hyperlipidemia of genetic origin [205] and improved intracellular cholesterol distribution in Kupffer cells in LDLR (low-density lipoprotein receptor) knockout mice on a high-fat high-cholesterol diet [206]. Intraperitoneal application of MβCD resulted in normalized serum lipid profiles, reduced aortic plaque lesions and decreased intraplaque inflammation in apolipoprotein E-deficient mice on a high-cholesterol diet [201]. Similar effects were found in New Zealand white rabbits on a high-fat diet after oral HPβCD [207]. In apolipoprotein E-knockout mice on a cholesterol-rich diet HPβCD applied subcutaneously solubilized extra- and intracellular cholesterol crystals in macrophages, prevented the formation of atherosclerotic lesions in the aorta and even induced regression of established advanced plaques. These effects were accompanied by complex liver X receptor-dependent transcriptional reprogramming of macrophages leading to their stimulated reverse cholesterol transport and attenuated inflammatory phenotype [208]. While exact mechanisms of CD-induced effects in atherosclerosis and their applicability in the human disease are unknown, promising results of these preclinical studies propose CDs as potentially efficient therapeutic alternatives in the disease. An encouraging direction of development is represented by nanoscopic CD formulations, such as luminol-conjugated βCD with good tolerability and efficient cellular internalization through the endo-lysosomal system, which was shown to efficiently inhibit proinflammatory cytokine production of macrophages in cellular studies and alleviate plaque injury and inflammation in a mouse model [209]. In apolipoprotein E-deficient mice, βCD polymers with a diameter of approximately 10 nm showed superior characteristics when compared to monomeric HPβCD in terms of better pharmacokinetics, plaque targeting and tolerability [210]. Affinity-driven cargo-switching nanoparticles composed of a core made of an MβCD-simvastatin inclusion complex and a shell of phospholipids demonstrated improved pharmacokinetics, more extensive colocalization with macrophages and cholesterol crystals in plaques, better clinical efficacy to dissolve cholesterol crystals and reduce plaque growth, and in cases of chronic application, even dissolution of existing lesions [211].

### 4.4. Cyclodextrins as Active Cholesterol-Extracting Agents and Direct Modulators of Protein Function in Niemann–Pick Type C Disease

Niemann–Pick type C is a rare, monogenic lysosomal storage disease characterized by the accumulation of free unesterified cholesterol especially in the late endosomes and lysosomes (LE/LY) of cells in both peripheral tissues (mostly liver, spleen and lung) and in the central nervous system typically leading to progressive neurodegeneration, severe neurological symptoms and eventually an early death. The disorder is caused by loss-of-function mutations in NPC1 or NPC2 proteins normally involved in the transfer of free cholesterol out of LE/LY towards the endoplasmic reticulum or the cell membrane. The lack of NPC1 or NPC2 activity results in the disruption of intracellular trafficking and consequent accumulation of free unesterified cholesterol mainly in LE/LY, which is accompanied by enhanced cholesterol uptake and endogenous synthesis [212,213,214]. Cellular defect of autophagy represents another characteristic alteration associated with Niemann–Pick type C disease, which typically manifests as impaired autophagosome–lysosome fusion and consequent accumulation of autophagosomes contributing to neuronal damage [155,215].

Given that cholesterol accumulation is pathognomic for Niemann–Pick type C and βCDs can efficiently form inclusion complexes with cholesterol it is reasonable to assume that these compounds can represent biologically relevant therapeutic tools by depleting the accumulated cholesterol in the disease [174]. βCD-mediated cholesterol extraction can occur through direct binding either at the plasma membrane leading to replenishment from intracellular pools eventually decreasing cholesterol levels in LE/LY, or directly in LE/LY after the cellular entry of βCDs via pinocytosis or others form of endocytosis. Consistent with this hypothesis, studies using cellular Niemann–Pick type C models demonstrated beneficial effects of βCDs including HPβCD and MβCD typically applied at concentrations between 100 µM and 1 mM. In human fibroblast cell lines with NPC1 or NPC2 deficiency, HPβCD and more potently MβCD, decreased LE/LY cholesterol accumulation and experiments suggested that cholesterol complexation by these βCDs mainly occurs in LE/LY and not in the plasma membrane [216]. Similarly, MβCD and HPβCD ameliorated lysosomal cholesterol accumulation in primary cultured neurons and glial cells from NPC1 mutant mice [217], human induced pluripotent cells carrying NPC1 mutation differentiated towards neural stem cells [218] and hepatic cells [219]. Based on experimental results, βCDs can actually act as direct shuttle for cholesterol towards the endoplasmic reticulum for esterification [220] or to the cell membrane and subsequently to the extracellular space [221], thereby filling in the role of mutant NPC1 or NPC2.

While the above-mentioned studies proposed cholesterol complexation as the principal mechanism of βCD action in Niemann–Pick type C disease, subsequent reports demonstrated that these compounds could activate alternative signaling pathways through the direct modulation of protein function, which finally result in decreasing lysosomal cholesterol levels [174]. One such mechanism can be the restoration of impaired autophagy flux. In NPC1 patient-derived fibroblasts, and induced pluripotent stem cells differentiated into neurons, 100 µM MβCD was found to activate AMPK via direct binding to its β subunits as discussed in detail in Section 3.4, which in turn activated downstream signaling and restored the impaired autophagosome–lysosome fusion and autophagy flux. These changes were accompanied by decreases in cellular cholesterol levels, which may also partially result from AMPK activation-induced ABCA1 (ATP binding cassette transporter A1)-mediated cholesterol efflux or inhibition of sterol-regulatory element-binding proteins (SREBPs). MβCD-induced cholesterol reduction was nearly abolished after knockdown of AMPK β subunits or treatment with AMPK inhibitor, while it was mimicked by AMPK activators, supporting the central role of AMPK in MβCD effects [155]. Another mechanism related to βCD effects on proteins was demonstrated by cellular studies in which 700 µM HPβCD induced cholesterol secretion from LE/LY into the extracellular space via a mechanism requiring the activation of MCOLN-1 (mucolipin-1 or TRPML1 (transient receptor potential mucolipin 1)) lysosomal calcium channels [222]. A recent study using NPC1-deficient cells and a metabolically traceable cholesterol derivative followed the route of the exogenously applied cholesterol and suggested that the lysosomal cholesterol secretion induced by HPβCD applied at the concentration of 500 µM involves trafficking to the plasma membrane and a transfer of cholesterol to extracellular lipoproteins. In the absence of these acceptors, cholesterol can be rather re-esterified in the endoplasmic reticulum [223]. Recently, proteomic comparison of NPC1 patient-derived and wild-type fibroblasts identified the lysosome-associated membrane protein 1 (LAMP-1) as a target of CD action since it was induced by 1 mM HPβCD and suggested to replace the function of mutant NPC1 protein to facilitate cholesterol export from the LE/LY compartments [224]. As demonstrated by these studies, additional mechanisms of action involving direct protein targets might synergistically complement cholesterol sequestering effects of βCDs to alleviate NPC1 alterations; however, such processes are not taken into consideration in human clinical studies. Interestingly, another CD derivative, HPγCD applied at 1 mM can reduce cholesterol levels in experimental models of NPC [224,225,226] in spite of its much lower ability to form inclusion complexes with cholesterol [11]. Furthermore, in NPC1 patient-derived fibroblasts 1 mM HPγCD (but not HPβCD) was able to activate TFEB, a master regulator of lysosomal functions, which resulted in an increase in lysosome–endoplasmic reticulum association and simultaneously enhanced the impaired autophagy-lysosomal pathway [227]. Favorable effects of HPγCD further underlines the importance of protein-mediated actions of CD derivatives in Niemann–Pick type C disease.

The therapeutic potential of CDs was demonstrated not only in cellular Niemann–Pick type C models but in vivo studies as well. In such cases, HPβCD is more frequently applied because of its better in vivo tolerability. In animal Niemann–Pick type C models, HPβCD-induced decreases in lysosomal cholesterol levels, reduced neurodegeneration and concomitant improvements in lifespan, and delays in the occurrence of symptoms were demonstrated after a single subcutaneous dose [228,229], chronic subcutaneous [230,231,232], intraperitoneal [230,233] or intrathecal [233] administration, or a combined subcutaneous and intrathecal application [234,235]. Based on promising data obtained in cellular and animal studies, HPβCD recently became involved in many clinical trials. In the first clinical cases, it was applied intravenously at doses as high as 2500 mg/kg/week, however, without significant stable improvements in the neurological status due to poor penetration through the blood–brain barrier [236,237,238]. Therefore, subsequent studies applied intrathecal (via lumbar injection) and intracerebroventricular (via an Ommaya reservoir at a dose of 200 to 400 mg every two weeks) administrations to deliver HPβCD to the central nervous system and better results were observed [239,240]. Due to its effectiveness and the lack of an FDA-approved drug to treat Niemann–Pick type C disease in the United States, FDA granted an orphan drug status to HPβCD for the treatment of Niemann–Pick type C, and it is a current subject of various ongoing clinical trials examining the safety, tolerability, pharmacokinetics and efficacy of two different HPβCD formulations (Trappsol^®^ Cyclo™ or VTS-270 having degrees of substitutions of ~7 and ~4.3, respectively) to alleviate neural and hepatic manifestations (Table 2). The applicability of HPβCD is limited by its side effects including tinnitus, chemical meningitis, renal toxicity, psychiatric symptoms, fever and the most extensively studied adverse reaction, sensorineural hearing loss [238,241,242]. As demonstrated by animal studies, such side effects could be attenuated by the application of HPγCD, SBEβCD or SBEγCD; however, the therapeutic efficiency of these derivatives is smaller than that of HPβCD [231].

While current CDs and treatment regimes show promising results, further studies are required to enhance the potential of CDs to treat Niemann–Pick type C disease. Possible routes of development involve optimization of treatment protocols, for example, through combined intravenous and intrathecal administration [243], utilization of other βCD derivatives [244,245], application of CD macromolecules such as covalently linked derivatives [246], epichlorohydrin-derived, stable HPβCD crosslinks [247] or noncovalent tethering into a linear polymeric polyrotaxane chain [248], which can provide better pharmacokinetic profiles, more efficient penetration through the blood–brain barrier and even lysosomal targeting. Further understanding of the detailed molecular mechanisms of CD actions including their effects on pathophysiologically relevant protein functions would substantially help the design of more effective CDs and application schemes.

## 5. Conclusions

**Figure 6:**
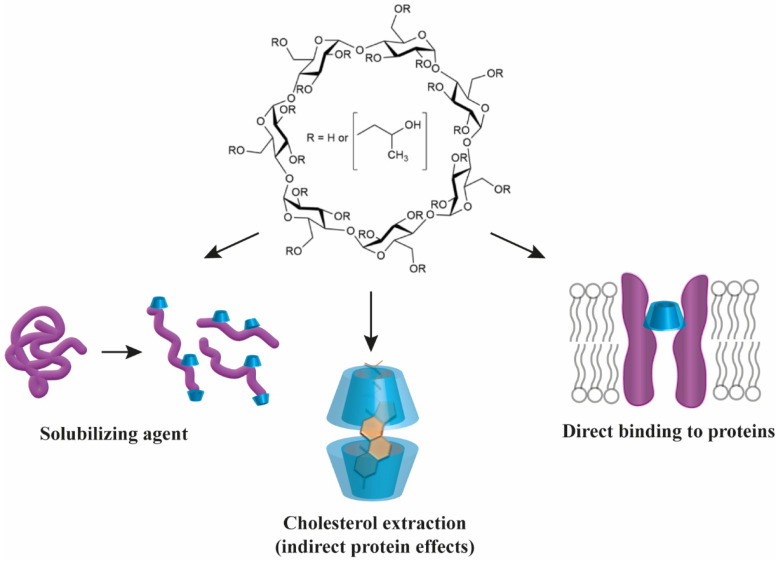
Potential therapeutic applications of cyclodextrins. HPβCD (upper panel), made of seven α-1,4-D-glucopyranoside units randomly hydroxypropylated at hydroxyl groups as indicated by R groups, is the most commonly applied βCD derivative in vivo. Potential functional effects of therapeutic CD applications (lower panels) are based on (i) anti-aggregative and solubilizing properties by shielding aggregation-prone hydrophobic groups of molecules; (ii) cholesterol extraction and consequent lipid raft disruption leading to an indirect modulation of protein function through alterations in biophysical parameters and lateral organization of bilayers; and (iii) direct binding to proteins at sites with a certain configuration of amino acid residues. While solubilizing actions are widely utilized in clinical practice, cholesterol-depleting abilities are mostly recognized in Niemann-Pick type C disease. Indirect actions on proteins associated with cholesterol extraction and direct, ligand-like CD–protein interactions are completely neglected in clinical practice in spite of in vitro and in vivo studies revealing general mechanisms and functional consequences of CD binding to proteins.

Due to their unique structure characterized by a hydrophilic outer surface and a hydrophobic cavity, CDs can associate with a variety of biological macromolecules, which provides the basis for their widespread industrial and pharmaceutical applications. While the molecular background of their utilization as excipients in drug formulations is generally well known, their potential use as active therapeutic agents is scarcely documented in spite of their ability to bind cholesterol and proteins. As summarized in Figure 6, favorable effects of CDs of potential therapeutic relevance can be based on three different mechanisms. First, CDs can form inclusion complexes with aggregation-prone hydrophobic groups of molecules resulting in solubilizing and anti-aggregative effects that can be utilized in peptide and protein drug formulations or in amyloid-related disorders such as Alzheimer’s or Parkinson’s disease. Second, through complexation, CDs can deplete membrane cholesterol. Based on this cholesterol-complexing ability, HPβCD was the first CD applied as an FDA approved active agent under the designation of an orphan drug in the therapy of Niemann–Pick type C disease. In parallel with cholesterol depletion, the disruption of lipid raft microdomains serving as signaling platforms can result in substantial alterations in biophysical parameters and lateral organization of the cell membrane, which can in turn indirectly modify the functional activity of proteins. However, such mechanisms of action represent currently completely unexploited therapeutic aspects of CDs. Third, CDs can directly bind proteins having an appropriate configuration of amino acids suitable for forming a CD binding site, such as those in AMPK, amyloid-forming β-amyloid peptides and α-synuclein, or pore-forming connexin, TASK-1 and K_V_1.3 channels. These direct CD–protein associations are generally mediated by a stacking inclusion between the CD inner cavity and an interacting central aromatic residue, and a network of hydrogen bonds, hydrophobic and van der Waals interactions with certain amino acids with appropriate configuration in sterical proximity in the three-dimensional structure. These direct effects can be biologically relevant even in pharmaceutical applications of CDs as excipients or therapeutically active agents in Niemann–Pick type C disease when local concentrations in the millimolar range are reached. Such millimolar CD levels can modify protein functions, as demonstrated in AMPK, TFEB and channels including connexin, K_V_1.3 and TASK. While anti-aggregative and membrane cholesterol-lowering properties are recognized to a certain degree in the literature of human diseases, indirect and direct mechanisms of protein function modulation are almost completely neglected by such studies. While CDs are considered well-tolerated and safe agents in vivo, still unexplored direct protein effects can not only be therapeutically beneficial, but they can even contribute to side effects as off-target actions, which could hinder their autonomous therapeutic use potentially necessitating the development of selective CD derivatives. Examples of CD-mediated functional effects on proteins collected in this review suggest that deeper understanding of molecular patterns of direct and indirect CD–protein interactions can reveal currently unexplored protein targets and pathological conditions where CDs can be efficiently utilized.

## Figures and Tables

**Table 1:** Cyclodextrin effects on human pore-forming proteins mediated by direct binding.

Protein	Cyclodextrin	Concentration	Method	Intramolecular Target	Findings
Connexin	αCD, βCD, γCD	5–20 mM	Transport-specific fractionation of unilamellar liposomes	Pore (specific residues not determined)	Reversible complete pore block in sucrose and urea permeation Characteristics of block depends on the size of CD relative to pore diameter
GABA_A_ receptor	βCD	0.15–1.5 mM	Patch-clamp	Not examined	Increased ligand-induced conductance Decelerated deactivation kinetics Decreased rate and extent of desensitization
TASK-1, TASK-3	αCD, MβCD	5 mM	Patch-clamp, molecular docking	Residues in the extracellular cavity close to the entrance of the pore (A chain: Arg68 and Gly97; B chain: Glu37, Lys70, Trp184, Gly203, Asp204 and Lys210)	Ionic currents reduced by ~40% Cholesterol-complexing filipin exerted no effects Direct binding between MβCD or αCD and the channel
K_V_1.3	MβCD, iαCD, iγCD	1–5 mM	Patch-clamp, molecular docking	Residues at the extracellular entrance of the pore (Thr372, Gly396, Asp397 and His399 of one subunit; Gly396 and His399 of one neighboring subunit; Gly396 and Asp397 of the opposing subunit)	Dose-dependent and partially reversible ~40% current reduction, apparent within 15 s and completed in 90 s Cholesterol-depleting HPβCD and HPγCD exerted no effects No correlation with changes in membrane fluidity, hydration or lipid order Direct binding between MβCD and the channel

**Table 2:** Clinical trials registered at clinicaltrials.gov examining the safety, tolerability, pharmacokinetics and efficacy of HPβCD to alleviate neural and hepatic manifestations of Niemann–Pick type C disease.

Identifier	Phase	Participants	Status	Type	Drug	Dosing
NCT01747135	1	14	Completed	Non-randomized, open-label, single-center	HPβCD (VTS-270 /Adrabetadex)	Intrathecal lumbar injection monthly 200 mg escalated to 900 mg
NCT02534844	2/3	51	Active, not recruiting	Prospective, randomized, double-blind, placebo controlled, multi-center	HPβCD (VTS-270 /Adrabetadex)	Intrathecal lumbar injection 900–1800 mg every 2 weeks
NCT02912793	1/2	12	Completed	Double-blind, randomized, multi-center	HPβCD (Trappsol^®^ Cyclo™)	Intravenous infusion 1500/2000/2500 mg/kg every 2 weeks
NCT02939547	1	13	Completed	double-blind, randomized, multi-center	HPβCD (Trappsol^®^ Cyclo™)	Intravenous infusion 1500/2500 mg/kg every 2 weeks
NCT03471143	1/2	3	Active, not recruiting	Open-label, dose escalation, multi-center	HPβCD (VTS-270 /Adrabetadex)	Intravenous infusion 500/1000 mg/kg twice a week for 6 weeks
NCT03687476	2	0	Withdrawn (business decision)	Open-label, multi-center	HPβCD (VTS-270 /Adrabetadex)	Intrathecal lumbar injection 200 mg escalated to 900 mg every 2 weeks
NCT03887533	1/2	2	Terminated (due to COVID-19)	Open-label, randomized, parallel dose, single-center	HPβCD (VTS-270 /Adrabetadex)	Combined intravenous infusion 500/1000 mg/kg monthly + intrathecal lumbar injection 900 mg monthly
NCT03893071	1	12	Active, not recruiting	Open-label extension study of NCT02939547	HPβCD (Trappsol^®^ Cyclo™)	Intravenous infusion 1500/2500 mg/kg every 2 weeks
NCT04860960	3	93	Recruiting	Prospective, randomized, double-blind, placebo controlled, multi-center	HPβCD (Trappsol^®^ Cyclo™)	Intravenous infusion 2000 mg/kg every 2 weeks
